# Crystal structure of *N*-[(4-eth­oxy­phen­yl)carbamo­thio­yl]cyclo­hexa­ne­carboxamide

**DOI:** 10.1107/S205698901501806X

**Published:** 2015-10-07

**Authors:** G. Vimala, J. Haribabu, S. Srividya, R. Karvembu, A. SubbiahPandi

**Affiliations:** a Department of Physics, Presidency College (Autonomous), Chennai 600 005, India; b Department of Chemistry, National Institute of Technology, Trichy 620 015, India

**Keywords:** crystals structure, thio­urea derivatives, biological properties, anti­corrosion properties, cyclo­hexa­necarboxamide, C—H⋯π inter­actions

## Abstract

The asymmetric unit of the title compound, C_16_H_22_N_2_O_2_S, contains two crystallographically independent mol­ecules (*A* and *B*). In mol­ecule *A*, the cyclo­hexane ring is disordered over two orientations [occupancy ratio 0.841 (10):0.159 (10)]. In each mol­ecule, the central carbonyl thio­urea unit is nearly planar (r.m.s. deviations for all non-H atoms of 0.034 Å in mol­ecule *A* and 0.094 Å in mol­ecule *B*). In both mol­ecules, the cyclo­hexane ring adopts a chair conformation. The mean plane of the cyclo­hexane ring makes dihedral angles of 35.8 (4) (mol­ecule *A*) and 20.7 (3)° (mol­ecule *B*) with that of the benzene ring. Each mol­ecule features an intra­molecular N—H⋯O hydrogen bond, which closes an *S*(6) ring motif. In the crystal, mol­ecules are linked *via* pairs of weak N—H⋯S inter­actions, forming inversion dimers with an *R*_2_^2^(8) ring motif for both mol­ecules. The crystal structure also features weak C—H⋯π ring inter­actions.

## Related literature   

For the biological and anti­corrosion properties of thio­urea derivatives, see: Hu *et al.* (2011 ▸); Sun *et al.* (2006 ▸); Shen *et al.* (2006 ▸). For related structure see: Vimala *et al.* (2015 ▸); Gangadharan *et al.* (2015 ▸).
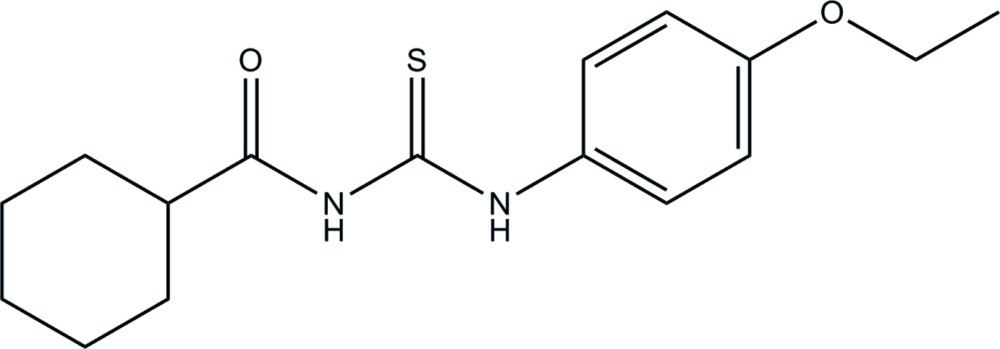


## Experimental   

### Crystal data  

C_16_H_22_N_2_O_2_S
*M* *_r_* = 306.41
Triclinic 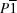

*a* = 10.2273 (7) Å
*b* = 12.0946 (7) Å
*c* = 15.2099 (10) Å
α = 70.792 (3)°
β = 89.100 (3)°
γ = 69.737 (3)°
*V* = 1656.42 (19) Å^3^
*Z* = 4
Mo *K*α radiation
μ = 0.20 mm^−1^
*T* = 293 K
0.30 × 0.20 × 0.20 mm

### Data collection  

Bruker Kappa APEXII CCD diffractometer
Absorption correction: multi-scan (*SADABS*; Bruker, 2004 ▸) *T*_min_ = 0.942, *T*_max_ = 0.961
35699 measured reflections
5827 independent reflections
3444 reflections with *I* > 2σ(*I*)
*R*_int_ = 0.049

### Refinement  

*R*[*F* ^2^ > 2σ(*F* ^2^)] = 0.060
*wR*(*F* ^2^) = 0.205
*S* = 1.10
5827 reflections
503 parameters
97 restraints
H atoms treated by a mixture of independent and constrained refinement
Δρ_max_ = 0.38 e Å^−3^
Δρ_min_ = −0.31 e Å^−3^

### 

Data collection: *APEX2* (Bruker, 2008 ▸); cell refinement: *APEX2* and *SAINT* (Bruker, 2008 ▸); data reduction: *SAINT* and *XPREP* (Bruker, 2008 ▸); program(s) used to solve structure: *SHELXS97* (Sheldrick, 2008 ▸); program(s) used to refine structure: *SHELXL2014* (Sheldrick, 2015 ▸); molecular graphics: *ORTEP-3 for Windows* (Farrugia, 2012 ▸); software used to prepare material for publication: *PLATON* (Spek, 2009 ▸).

## Supplementary Material

Crystal structure: contains datablock(s) global, I. DOI: 10.1107/S205698901501806X/jj2193sup1.cif

Structure factors: contains datablock(s) I. DOI: 10.1107/S205698901501806X/jj2193Isup2.hkl

Supporting information file. DOI: 10.1107/S205698901501806X/jj2193Isup3.cml

**Fig. 1.**
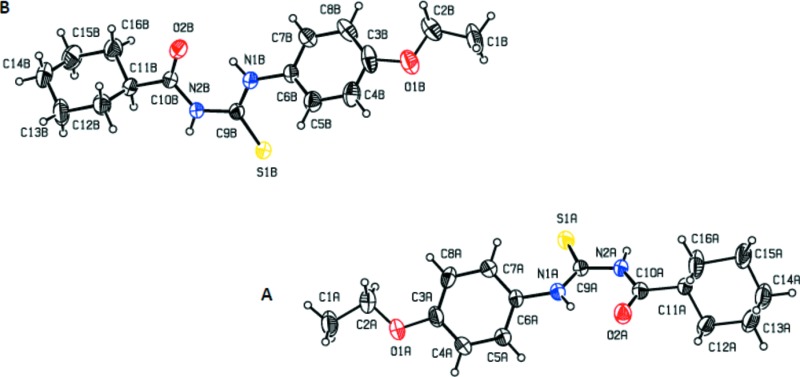
The molecular structure of the major component of the title compound, with displacement ellipsoids drawn at 30% probability level.

**Fig. 2.**
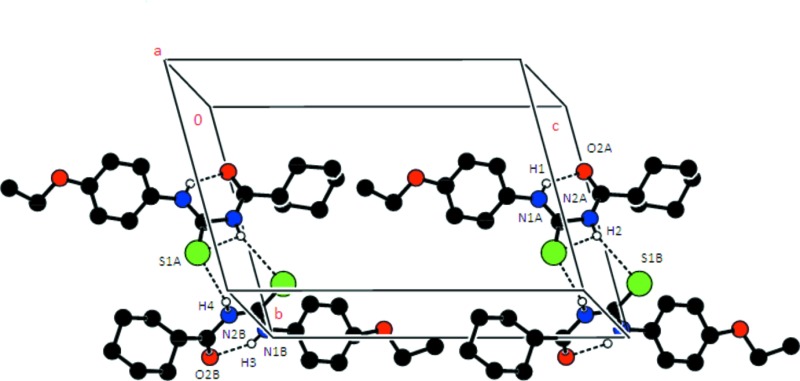
A view of the packing of (I) along the a axis, showing N—H···O intramolecular hydrogen bonds and molecules linked by weak N—H···S intermolecular interactions along the b axis.

. DOI: 10.1107/S205698901501806X/jj2193fig1.tif
The mol­ecular structure of the major component of the title compound, with displacement ellipsoids drawn at 30% probability level.

a b . DOI: 10.1107/S205698901501806X/jj2193fig2.tif
A view of the packing of (I) along the *a* axis, showing N—H⋯O intra­molecular hydrogen bonds and mol­ecules linked by weak N—H⋯S inter­molecular inter­actions along the *b* axis.

CCDC reference: 1427899

Additional supporting information:  crystallographic information; 3D view; checkCIF report

## Figures and Tables

**Table 1:** Hydrogen-bond geometry (, ) *Cg*1 is the centroid of the C3*A*C8*A* ring.

*D*H*A*	*D*H	H*A*	*D* *A*	*D*H*A*
C2*B*H2*D*O2*B* ^i^	0.97	2.57	3.264(10)	128
N1*B*H3O2*B*	0.86(2)	1.91(3)	2.641(4)	142(4)
N1*A*H1O2*A*	0.87(2)	1.90(3)	2.628(4)	140(4)
N2*B*H4S1*A* ^ii^	0.84(2)	2.68(2)	3.469(3)	157(3)
N2*A*H2S1*B* ^iii^	0.85(2)	2.73(3)	3.430(3)	140(3)
C12H12*C* *Cg*1^iv^	0.90	2.49(2)	3.42(1)	159

Symmetry codes: (i) 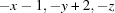; (ii) 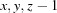; (iii) 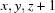; (iv) 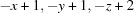.

## supplementary crystallographic information

### S1. Comment

The design and synthesis of thioureas are of considerable interest because of their use in agriculture, medicine and analytical chemistry (Hu *et al.*, 2011). Thiourea derivatives are driven by their potential as biological active compounds (Sun *et al.*, 2006) and in material applications such as with their anti corrosion prperties (Shen *et al.*, 2006). In view of their biological importance, the crystal structure of the title compound, C_16_H_22_N_2_O_2_S, (I), has been determined herein.

The title compound, (I), contains two crystallographically independent molecules (A and B) in the asymmetric unit (Fig1.). In molecule A, the cyclohexane ring is disordered over two positions [occupancy ratio 0.533 (2): 0.467 (2)]. In each molecule, the central carbonyl thiourea unit is nearly planar (r.m.s. deviations for all non-H atoms of -0.034 Å for C6A and -0.094 Å for C6B. For molecule A, the cyclohexane ring (C11A—C16A) adopts a chairconformation [puckering parameters, q = 0.627 (1) Å, θ = 6.8°, φ = 279 (2)°], while for molecule B, the cyclohexane ring (C11B—C16B) also adopts a chair conformation [puckering, q = 0.546 (6) Å, θ = 179.3°, φ = 219 (2)°;]. The mean plane of the cyclohexane ring makes a dihedral angle of 35.8 (4)° (C3A—C8A) and 20.7 (3)° (C3B—C8B) with that of the benzene ring. Each molecule features an intramolecular N—H···O hydrogen bond (Table 1), which closes an S(6) ring motif. In the crystal, the molecules are linked *via* pairs of N—H···S weak intermolecular interactions, forming inversion dimers with an *R*^2^_2_(8) ring motif (Bernstein *et al.* 1995) for both molecules (Fig. 2). The crystal structure is further stabilized by a weak C—H···π ring interactions (Table 1).

### S2. Experimental

A mixture of 6-chlorol-3-formylchromone (1 mmol), cyanoacetylindole (1 mmol) and ammonium acetate (1 mmol) in DMF and a catalytic amount of SnCl2.2H2O (0.020 mol %) was added and refluxed for about 3 hrs. After completition of the reaction, the solvent was removed under reduced pressure and the residue was purified by column chromatography on silica gel (3:97% ethylacetate and petether) to afford a pure product. The purified compound was recrystalized from ethanol by using the slow evaporation method. The yield of the isolated product was 92%, giving block-like crystals suitable for *X* ray diffraction.

### S3. Refinement

All H atoms were fixed geometrically and allowed to ride on their parent C atoms, with C—H distances fixed in the range 0.93–0.97 Å with *U*_iso_(H) = 1.5*U*_eq_(C) for methyl H atoms and 1.2*U*_eq_(C) for all other H atoms.

### Crystal data

**Table:**

C_16_H_22_N_2_O_2_S	*Z* = 4
*M**_r_* = 306.41	*F*(000) = 656
Triclinic, *P*1	*D*_x_ = 1.229 Mg m^−^^3^
*a* = 10.2273 (7) Å	Mo *K*α radiation, λ = 0.71073 Å
*b* = 12.0946 (7) Å	Cell parameters from 3444 reflections
*c* = 15.2099 (10) Å	θ = 2.3–25.0°
α = 70.792 (3)°	µ = 0.20 mm^−^^1^
β = 89.100 (3)°	*T* = 293 K
γ = 69.737 (3)°	Block, colourless
*V* = 1656.42 (19) Å^3^	0.30 × 0.20 × 0.20 mm

### Data collection

**Table:**

Bruker Kappa APEXII CCD diffractometer	5827 independent reflections
Radiation source: fine-focus sealed tube	3444 reflections with *I* > 2σ(*I*)
Detector resolution: 8.33 pixels mm^-1^	*R*_int_ = 0.049
ω and φ scan	θ_max_ = 25.0°, θ_min_ = 2.3°
Absorption correction: multi-scan (*SADABS*; Bruker, 2004)	*h* = −12→12
*T*_min_ = 0.942, *T*_max_ = 0.961	*k* = −14→14
35699 measured reflections	*l* = −18→18

### Refinement

**Table:**

Refinement on *F*^2^	Primary atom site location: structure-invariant direct methods
Least-squares matrix: full	Secondary atom site location: difference Fourier map
*R*[*F*^2^ > 2σ(*F*^2^)] = 0.060	Hydrogen site location: mixed
*wR*(*F*^2^) = 0.205	H atoms treated by a mixture of independent and constrained refinement
*S* = 1.10	*w* = 1/[σ^2^(*F*_o_^2^) + (0.0708*P*)^2^ + 1.8566*P*] where *P* = (*F*_o_^2^ + 2*F*_c_^2^)/3
5827 reflections	(Δ/σ)_max_ = 0.008
503 parameters	Δρ_max_ = 0.38 e Å^−^^3^
97 restraints	Δρ_min_ = −0.31 e Å^−^^3^

### Special details

**Table:**

Geometry. All e.s.d.'s (except the e.s.d. in the dihedral angle between two l.s. planes) are estimated using the full covariance matrix. The cell e.s.d.'s are taken into account individually in the estimation of e.s.d.'s in distances, angles and torsion angles; correlations between e.s.d.'s in cell parameters are only used when they are defined by crystal symmetry. An approximate (isotropic) treatment of cell e.s.d.'s is used for estimating e.s.d.'s involving l.s. planes.

### Fractional atomic coordinates and isotropic or equivalent isotropic displacement parameters (Å^2^)

**Table:**

	*x*	*y*	*z*	*U*_iso_*/*U*_eq_	Occ. (<1)
C3A	0.6065 (5)	0.4664 (5)	0.6568 (3)	0.0699 (12)	
C4A	0.6435 (5)	0.3687 (4)	0.7381 (3)	0.0724 (12)	
H4A	0.7200	0.2965	0.7433	0.087*	
C5A	0.5696 (4)	0.3749 (4)	0.8131 (3)	0.0641 (11)	
H5A	0.5961	0.3065	0.8687	0.077*	
C6A	0.4571 (4)	0.4806 (4)	0.8074 (3)	0.0538 (9)	
C7A	0.4174 (5)	0.5790 (4)	0.7246 (3)	0.0747 (13)	
H7A	0.3398	0.6505	0.7191	0.090*	
C8A	0.4926 (5)	0.5725 (5)	0.6489 (3)	0.0802 (14)	
H8A	0.4661	0.6400	0.5927	0.096*	
C9A	0.3402 (4)	0.5683 (3)	0.9253 (2)	0.0513 (9)	
C10A	0.2564 (4)	0.4308 (4)	1.0544 (3)	0.0568 (10)	
C11A	0.1943 (6)	0.4272 (7)	1.1454 (4)	0.0594 (14)	0.841 (10)
H11A	0.1917	0.5013	1.1595	0.071*	0.841 (10)
C12A	0.2760 (7)	0.3116 (8)	1.2256 (4)	0.091 (2)	0.841 (10)
H12A	0.3716	0.3079	1.2331	0.109*	0.841 (10)
H12B	0.2788	0.2379	1.2120	0.109*	0.841 (10)
C13A	0.2113 (7)	0.3106 (9)	1.3156 (4)	0.102 (2)	0.841 (10)
H13A	0.2632	0.2328	1.3655	0.122*	0.841 (10)
H13B	0.2169	0.3796	1.3327	0.122*	0.841 (10)
C14A	0.0626 (13)	0.3229 (14)	1.3048 (7)	0.101 (3)	0.841 (10)
H14A	0.0224	0.3236	1.3629	0.121*	0.841 (10)
H14B	0.0575	0.2513	1.2916	0.121*	0.841 (10)
C15A	−0.0204 (7)	0.4420 (9)	1.2265 (4)	0.111 (3)	0.841 (10)
H15A	−0.0160	0.5146	1.2386	0.134*	0.841 (10)
H15B	−0.1179	0.4502	1.2204	0.134*	0.841 (10)
C16A	0.0492 (7)	0.4315 (11)	1.1333 (5)	0.127 (4)	0.841 (10)
H16A	0.0498	0.3560	1.1235	0.153*	0.841 (10)
H16B	−0.0037	0.5035	1.0794	0.153*	0.841 (10)
C11'	0.157 (3)	0.446 (5)	1.1254 (18)	0.0594 (14)	0.159 (10)
H11'	0.1460	0.5334	1.1146	0.071*	0.159 (10)
C12'	0.249 (3)	0.394 (4)	1.2150 (19)	0.080 (9)	0.159 (10)
H12C	0.3113	0.4397	1.2124	0.096*	0.159 (10)
H12D	0.3051	0.3061	1.2282	0.096*	0.159 (10)
C13'	0.148 (4)	0.410 (4)	1.291 (3)	0.095 (10)	0.159 (10)
H13C	0.2041	0.3842	1.3503	0.114*	0.159 (10)
H13D	0.0920	0.4985	1.2748	0.114*	0.159 (10)
C14'	0.049 (6)	0.337 (5)	1.306 (3)	0.072 (9)	0.159 (10)
H14C	0.1025	0.2479	1.3339	0.087*	0.159 (10)
H14D	−0.0174	0.3617	1.3485	0.087*	0.159 (10)
C15'	−0.029 (3)	0.363 (2)	1.213 (2)	0.070 (7)	0.159 (10)
H15C	−0.0033	0.2885	1.1965	0.084*	0.159 (10)
H15D	−0.1297	0.3958	1.2151	0.084*	0.159 (10)
C16'	0.020 (3)	0.461 (2)	1.147 (2)	0.056 (5)	0.159 (10)
H16C	−0.0363	0.4892	1.0878	0.068*	0.159 (10)
H16D	−0.0100	0.5314	1.1695	0.068*	0.159 (10)
C3B	−0.4223 (6)	0.9039 (7)	0.1897 (4)	0.0888 (16)	
C4B	−0.3314 (6)	0.7967 (5)	0.1788 (3)	0.0853 (15)	
H4B	−0.3251	0.7187	0.2209	0.102*	
C5B	−0.2503 (5)	0.8033 (4)	0.1071 (3)	0.0706 (12)	
H5B	−0.1893	0.7299	0.1000	0.085*	
C6B	−0.2580 (4)	0.9180 (4)	0.0450 (3)	0.0543 (9)	
C7B	−0.3527 (4)	1.0252 (4)	0.0530 (3)	0.0680 (11)	
H7B	−0.3611	1.1029	0.0096	0.082*	
C8B	−0.4358 (5)	1.0185 (5)	0.1255 (4)	0.0831 (14)	
H8B	−0.5007	1.0915	0.1307	0.100*	
C9B	−0.0343 (4)	0.8755 (3)	−0.0218 (2)	0.0530 (9)	
C10B	−0.0317 (5)	0.9956 (4)	−0.1891 (3)	0.0596 (10)	
C11B	0.0708 (4)	1.0160 (4)	−0.2589 (3)	0.0645 (11)	
H11B	0.1621	0.9908	−0.2238	0.077*	
C12B	0.0870 (6)	0.9341 (4)	−0.3166 (3)	0.0835 (14)	
H12E	−0.0036	0.9536	−0.3488	0.100*	
H12F	0.1195	0.8470	−0.2757	0.100*	
C13B	0.1893 (7)	0.9515 (6)	−0.3879 (4)	0.118 (2)	
H13E	0.2826	0.9221	−0.3559	0.142*	
H13F	0.1914	0.9017	−0.4270	0.142*	
C14B	0.1479 (7)	1.0875 (6)	−0.4484 (4)	0.111 (2)	
H14E	0.2193	1.0971	−0.4899	0.133*	
H14F	0.0605	1.1132	−0.4868	0.133*	
C15B	0.1305 (7)	1.1697 (5)	−0.3922 (4)	0.1058 (19)	
H15E	0.0968	1.2566	−0.4337	0.127*	
H15F	0.2210	1.1518	−0.3607	0.127*	
C16B	0.0288 (6)	1.1527 (4)	−0.3196 (3)	0.0879 (15)	
H16E	0.0279	1.2023	−0.2806	0.106*	
H16F	−0.0651	1.1823	−0.3508	0.106*	
N1A	0.3837 (3)	0.4788 (3)	0.8879 (2)	0.0570 (8)	
N2A	0.2747 (3)	0.5405 (3)	1.0059 (2)	0.0529 (8)	
N1B	−0.1730 (3)	0.9287 (3)	−0.0299 (2)	0.0571 (8)	
N2B	0.0275 (4)	0.9065 (3)	−0.1033 (2)	0.0576 (9)	
O2A	0.2912 (3)	0.3411 (3)	1.0280 (2)	0.0735 (8)	
O2B	−0.1578 (3)	1.0514 (3)	−0.20668 (19)	0.0787 (9)	
S1A	0.36072 (12)	0.70593 (10)	0.88640 (8)	0.0664 (3)	
S1B	0.06988 (12)	0.78100 (10)	0.07648 (7)	0.0643 (3)	
C1A	0.7842 (10)	0.5119 (10)	0.4461 (6)	0.102 (3)	0.867 (13)
H1A	0.8710	0.5065	0.4736	0.153*	0.867 (13)
H1B	0.7622	0.5724	0.3838	0.153*	0.867 (13)
H1C	0.7928	0.4313	0.4439	0.153*	0.867 (13)
C2A	0.6698 (8)	0.5516 (6)	0.5040 (4)	0.087 (2)	0.867 (13)
H2A	0.6723	0.6239	0.5174	0.104*	0.867 (13)
H2B	0.5790	0.5741	0.4707	0.104*	0.867 (13)
O1A	0.6919 (7)	0.4496 (5)	0.5875 (3)	0.089 (2)	0.867 (13)
C1A'	0.800 (13)	0.453 (7)	0.457 (6)	0.24 (7)	0.133 (13)
H1'1	0.8898	0.3905	0.4595	0.354*	0.133 (13)
H1'2	0.7327	0.4499	0.4161	0.354*	0.133 (13)
H1'3	0.8071	0.5348	0.4351	0.354*	0.133 (13)
C2A'	0.754 (4)	0.429 (5)	0.554 (4)	0.12 (2)	0.133 (13)
H2'1	0.7514	0.3448	0.5758	0.141*	0.133 (13)
H2'2	0.8256	0.4294	0.5947	0.141*	0.133 (13)
O1A'	0.625 (3)	0.512 (4)	0.5633 (14)	0.113 (19)	0.133 (13)
C1B	−0.608 (2)	0.913 (3)	0.3917 (12)	0.162 (10)	0.705 (13)
H1D	−0.6418	0.8479	0.3911	0.243*	0.705 (13)
H1E	−0.6811	0.9766	0.4070	0.243*	0.705 (13)
H1F	−0.5282	0.8774	0.4377	0.243*	0.705 (13)
C2B	−0.5672 (9)	0.9695 (9)	0.2959 (8)	0.096 (3)	0.705 (13)
H2D	−0.6494	1.0189	0.2505	0.115*	0.705 (13)
H2E	−0.5159	1.0228	0.2981	0.115*	0.705 (13)
O1B	−0.4816 (6)	0.8658 (7)	0.2729 (5)	0.094 (2)	0.705 (13)
O1B'	−0.5264 (13)	0.9542 (12)	0.2458 (8)	0.077 (5)	0.295 (13)
C1B'	−0.622 (3)	0.897 (6)	0.391 (2)	0.094 (12)	0.295 (13)
H1G	−0.7127	0.8934	0.3821	0.141*	0.295 (13)
H1H	−0.6312	0.9823	0.3809	0.141*	0.295 (13)
H1I	−0.5803	0.8455	0.4542	0.141*	0.295 (13)
C2B'	−0.530 (2)	0.8499 (16)	0.3233 (12)	0.076 (6)	0.295 (13)
H2F	−0.4355	0.7989	0.3542	0.091*	0.295 (13)
H2G	−0.5661	0.7982	0.3015	0.091*	0.295 (13)
H3	−0.208 (4)	0.983 (3)	−0.0843 (17)	0.064 (12)*	
H1	0.373 (4)	0.409 (2)	0.920 (3)	0.069 (13)*	
H4	0.115 (2)	0.868 (3)	−0.094 (2)	0.052 (11)*	
H2	0.259 (7)	0.612 (3)	1.011 (4)	0.18 (3)*	

### Atomic displacement parameters (Å^2^)

**Table:**

	*U*^11^	*U*^22^	*U*^33^	*U*^12^	*U*^13^	*U*^23^
C3A	0.073 (3)	0.086 (3)	0.056 (3)	−0.026 (3)	0.024 (2)	−0.036 (2)
C4A	0.069 (3)	0.072 (3)	0.065 (3)	−0.008 (2)	0.021 (2)	−0.028 (2)
C5A	0.072 (3)	0.057 (2)	0.057 (2)	−0.016 (2)	0.017 (2)	−0.019 (2)
C6A	0.060 (2)	0.059 (2)	0.043 (2)	−0.0187 (19)	0.0148 (18)	−0.0212 (18)
C7A	0.076 (3)	0.071 (3)	0.055 (3)	−0.002 (2)	0.012 (2)	−0.020 (2)
C8A	0.107 (4)	0.083 (3)	0.040 (2)	−0.029 (3)	0.015 (2)	−0.013 (2)
C9A	0.048 (2)	0.054 (2)	0.044 (2)	−0.0123 (17)	0.0081 (17)	−0.0143 (17)
C10A	0.062 (2)	0.053 (2)	0.051 (2)	−0.0176 (19)	0.0136 (19)	−0.0161 (19)
C11A	0.070 (3)	0.053 (3)	0.044 (3)	−0.015 (3)	0.017 (2)	−0.011 (3)
C12A	0.078 (3)	0.099 (4)	0.058 (3)	−0.018 (3)	0.013 (2)	0.005 (3)
C13A	0.099 (4)	0.122 (6)	0.054 (3)	−0.036 (4)	0.016 (3)	0.003 (3)
C14A	0.105 (5)	0.134 (7)	0.057 (4)	−0.051 (4)	0.029 (3)	−0.019 (3)
C15A	0.076 (4)	0.164 (7)	0.063 (3)	−0.033 (4)	0.033 (3)	−0.012 (3)
C16A	0.064 (4)	0.242 (10)	0.049 (3)	−0.045 (4)	0.019 (3)	−0.029 (4)
C11'	0.070 (3)	0.053 (3)	0.044 (3)	−0.015 (3)	0.017 (2)	−0.011 (3)
C12'	0.084 (9)	0.10 (2)	0.045 (5)	−0.045 (9)	0.011 (5)	0.000 (7)
C13'	0.124 (14)	0.13 (2)	0.074 (10)	−0.095 (16)	0.046 (10)	−0.039 (12)
C14'	0.073 (15)	0.063 (18)	0.087 (12)	−0.038 (14)	0.032 (9)	−0.020 (9)
C15'	0.068 (12)	0.044 (11)	0.093 (12)	−0.014 (9)	0.024 (8)	−0.024 (9)
C16'	0.070 (5)	0.037 (9)	0.071 (12)	−0.014 (5)	0.024 (5)	−0.036 (8)
C3B	0.083 (3)	0.147 (5)	0.078 (3)	−0.071 (4)	0.045 (3)	−0.062 (4)
C4B	0.097 (4)	0.106 (4)	0.070 (3)	−0.061 (3)	0.033 (3)	−0.027 (3)
C5B	0.082 (3)	0.070 (3)	0.067 (3)	−0.035 (2)	0.021 (2)	−0.026 (2)
C6B	0.059 (2)	0.061 (2)	0.047 (2)	−0.0248 (19)	0.0162 (18)	−0.0217 (19)
C7B	0.062 (3)	0.071 (3)	0.074 (3)	−0.021 (2)	0.018 (2)	−0.032 (2)
C8B	0.061 (3)	0.107 (4)	0.105 (4)	−0.030 (3)	0.032 (3)	−0.067 (3)
C9B	0.066 (3)	0.046 (2)	0.045 (2)	−0.0159 (18)	0.0125 (18)	−0.0172 (17)
C10B	0.066 (3)	0.057 (2)	0.043 (2)	−0.012 (2)	0.0104 (19)	−0.0114 (18)
C11B	0.065 (3)	0.070 (3)	0.042 (2)	−0.014 (2)	0.0096 (19)	−0.0082 (19)
C12B	0.102 (4)	0.074 (3)	0.071 (3)	−0.031 (3)	0.030 (3)	−0.024 (3)
C13B	0.166 (6)	0.111 (4)	0.080 (4)	−0.051 (4)	0.069 (4)	−0.038 (3)
C14B	0.143 (5)	0.117 (5)	0.058 (3)	−0.045 (4)	0.038 (3)	−0.014 (3)
C15B	0.129 (5)	0.094 (4)	0.087 (4)	−0.057 (4)	0.037 (4)	−0.006 (3)
C16B	0.118 (4)	0.076 (3)	0.072 (3)	−0.043 (3)	0.027 (3)	−0.020 (3)
N1A	0.065 (2)	0.053 (2)	0.0495 (19)	−0.0166 (17)	0.0179 (16)	−0.0186 (16)
N2A	0.0588 (19)	0.0508 (19)	0.0436 (17)	−0.0186 (15)	0.0152 (15)	−0.0112 (15)
N1B	0.056 (2)	0.062 (2)	0.0442 (19)	−0.0158 (17)	0.0111 (16)	−0.0128 (16)
N2B	0.057 (2)	0.0558 (19)	0.0431 (18)	−0.0065 (16)	0.0137 (16)	−0.0110 (15)
O2A	0.102 (2)	0.0593 (17)	0.0655 (18)	−0.0327 (16)	0.0270 (17)	−0.0270 (15)
O2B	0.069 (2)	0.090 (2)	0.0521 (17)	−0.0147 (17)	0.0034 (15)	−0.0073 (15)
S1A	0.0728 (7)	0.0585 (6)	0.0632 (7)	−0.0220 (5)	0.0257 (5)	−0.0177 (5)
S1B	0.0715 (7)	0.0613 (6)	0.0449 (6)	−0.0107 (5)	0.0108 (5)	−0.0137 (5)
C1A	0.116 (6)	0.122 (8)	0.074 (4)	−0.049 (5)	0.048 (4)	−0.038 (5)
C2A	0.105 (5)	0.108 (5)	0.055 (4)	−0.043 (4)	0.026 (4)	−0.033 (3)
O1A	0.089 (6)	0.097 (4)	0.063 (3)	−0.015 (4)	0.035 (3)	−0.028 (3)
C1A'	0.37 (14)	0.12 (7)	0.25 (10)	−0.10 (7)	0.26 (10)	−0.10 (7)
C2A'	0.07 (3)	0.13 (5)	0.18 (7)	−0.02 (3)	0.02 (3)	−0.09 (5)
O1A'	0.045 (19)	0.21 (5)	0.08 (3)	−0.02 (2)	0.023 (16)	−0.07 (3)
C1B	0.198 (18)	0.169 (17)	0.119 (15)	−0.052 (15)	0.111 (12)	−0.069 (13)
C2B	0.073 (5)	0.119 (8)	0.111 (8)	−0.030 (5)	0.037 (5)	−0.064 (7)
O1B	0.096 (4)	0.102 (5)	0.099 (6)	−0.044 (4)	0.051 (4)	−0.049 (5)
O1B'	0.082 (9)	0.060 (8)	0.080 (9)	−0.018 (7)	0.049 (7)	−0.024 (7)
C1B'	0.046 (12)	0.14 (3)	0.07 (2)	−0.018 (15)	0.013 (12)	−0.027 (18)
C2B'	0.065 (12)	0.089 (14)	0.069 (12)	−0.032 (10)	0.020 (9)	−0.019 (10)

### Geometric parameters (Å, º)

**Table:**

C3A—C4A	1.346 (6)	C5B—H5B	0.9300
C3A—C8A	1.373 (6)	C6B—C7B	1.366 (5)
C3A—O1A	1.381 (6)	C6B—N1B	1.421 (5)
C3A—O1A'	1.383 (19)	C7B—C8B	1.379 (6)
C4A—C5A	1.366 (5)	C7B—H7B	0.9300
C4A—H4A	0.9300	C8B—H8B	0.9300
C5A—C6A	1.370 (5)	C9B—N1B	1.328 (5)
C5A—H5A	0.9300	C9B—N2B	1.382 (5)
C6A—C7A	1.364 (5)	C9B—S1B	1.661 (4)
C6A—N1A	1.423 (5)	C10B—O2B	1.217 (5)
C7A—C8A	1.384 (6)	C10B—N2B	1.371 (5)
C7A—H7A	0.9300	C10B—C11B	1.502 (5)
C8A—H8A	0.9300	C11B—C12B	1.494 (6)
C9A—N1A	1.326 (5)	C11B—C16B	1.512 (6)
C9A—N2A	1.387 (4)	C11B—H11B	0.9800
C9A—S1A	1.659 (4)	C12B—C13B	1.510 (6)
C10A—O2A	1.216 (4)	C12B—H12E	0.9700
C10A—N2A	1.366 (5)	C12B—H12F	0.9700
C10A—C11'	1.482 (17)	C13B—C14B	1.505 (7)
C10A—C11A	1.508 (6)	C13B—H13E	0.9700
C11A—C16A	1.479 (8)	C13B—H13F	0.9700
C11A—C12A	1.497 (7)	C14B—C15B	1.479 (8)
C11A—H11A	0.9800	C14B—H14E	0.9700
C12A—C13A	1.510 (7)	C14B—H14F	0.9700
C12A—H12A	0.9700	C15B—C16B	1.519 (7)
C12A—H12B	0.9700	C15B—H15E	0.9700
C13A—C14A	1.482 (11)	C15B—H15F	0.9700
C13A—H13A	0.9700	C16B—H16E	0.9700
C13A—H13B	0.9700	C16B—H16F	0.9700
C14A—C15A	1.506 (12)	N1A—H1	0.871 (19)
C14A—H14A	0.9700	N2A—H2	0.85 (2)
C14A—H14B	0.9700	N1B—H3	0.855 (19)
C15A—C16A	1.599 (9)	N2B—H4	0.841 (18)
C15A—H15A	0.9700	C1A—C2A	1.493 (9)
C15A—H15B	0.9700	C1A—H1A	0.9600
C16A—H16A	0.9700	C1A—H1B	0.9600
C16A—H16B	0.9700	C1A—H1C	0.9600
C11'—C16'	1.402 (18)	C2A—O1A	1.404 (7)
C11'—C12'	1.485 (19)	C2A—H2A	0.9700
C11'—H11'	0.9800	C2A—H2B	0.9700
C12'—C13'	1.555 (19)	C1A'—C2A'	1.49 (2)
C12'—H12C	0.9700	C1A'—H1'1	0.9600
C12'—H12D	0.9700	C1A'—H1'2	0.9600
C13'—C14'	1.53 (2)	C1A'—H1'3	0.9600
C13'—H13C	0.9700	C2A'—O1A'	1.40 (2)
C13'—H13D	0.9700	C2A'—H2'1	0.9700
C14'—C15'	1.51 (2)	C2A'—H2'2	0.9700
C14'—H14C	0.9700	C1B—C2B	1.515 (17)
C14'—H14D	0.9700	C1B—H1D	0.9600
C15'—C16'	1.505 (19)	C1B—H1E	0.9600
C15'—H15C	0.9700	C1B—H1F	0.9600
C15'—H15D	0.9700	C2B—O1B	1.408 (9)
C16'—H16C	0.9700	C2B—H2D	0.9700
C16'—H16D	0.9700	C2B—H2E	0.9700
C3B—C4B	1.369 (7)	O1B'—C2B'	1.426 (15)
C3B—C8B	1.370 (7)	C1B'—C2B'	1.51 (2)
C3B—O1B	1.403 (7)	C1B'—H1G	0.9600
C3B—O1B'	1.448 (12)	C1B'—H1H	0.9600
C4B—C5B	1.358 (6)	C1B'—H1I	0.9600
C4B—H4B	0.9300	C2B'—H2F	0.9700
C5B—C6B	1.373 (5)	C2B'—H2G	0.9700
			
C4A—C3A—C8A	119.7 (4)	C5B—C6B—N1B	121.8 (4)
C4A—C3A—O1A	114.8 (4)	C6B—C7B—C8B	120.2 (4)
C8A—C3A—O1A	125.5 (5)	C6B—C7B—H7B	119.9
C4A—C3A—O1A'	147.5 (17)	C8B—C7B—H7B	119.9
C8A—C3A—O1A'	92.4 (16)	C3B—C8B—C7B	119.7 (5)
C3A—C4A—C5A	120.5 (4)	C3B—C8B—H8B	120.2
C3A—C4A—H4A	119.7	C7B—C8B—H8B	120.2
C5A—C4A—H4A	119.7	N1B—C9B—N2B	115.7 (3)
C4A—C5A—C6A	120.9 (4)	N1B—C9B—S1B	126.3 (3)
C4A—C5A—H5A	119.6	N2B—C9B—S1B	117.9 (3)
C6A—C5A—H5A	119.6	O2B—C10B—N2B	121.7 (4)
C7A—C6A—C5A	118.8 (4)	O2B—C10B—C11B	123.5 (3)
C7A—C6A—N1A	123.6 (3)	N2B—C10B—C11B	114.7 (4)
C5A—C6A—N1A	117.5 (3)	C12B—C11B—C10B	110.1 (4)
C6A—C7A—C8A	120.1 (4)	C12B—C11B—C16B	111.5 (4)
C6A—C7A—H7A	119.9	C10B—C11B—C16B	111.7 (4)
C8A—C7A—H7A	119.9	C12B—C11B—H11B	107.8
C3A—C8A—C7A	119.9 (4)	C10B—C11B—H11B	107.8
C3A—C8A—H8A	120.1	C16B—C11B—H11B	107.8
C7A—C8A—H8A	120.1	C11B—C12B—C13B	112.0 (4)
N1A—C9A—N2A	115.5 (3)	C11B—C12B—H12E	109.2
N1A—C9A—S1A	126.3 (3)	C13B—C12B—H12E	109.2
N2A—C9A—S1A	118.2 (3)	C11B—C12B—H12F	109.2
O2A—C10A—N2A	122.9 (4)	C13B—C12B—H12F	109.2
O2A—C10A—C11'	123 (2)	H12E—C12B—H12F	107.9
N2A—C10A—C11'	112 (2)	C14B—C13B—C12B	110.8 (5)
O2A—C10A—C11A	122.2 (4)	C14B—C13B—H13E	109.5
N2A—C10A—C11A	114.9 (4)	C12B—C13B—H13E	109.5
C16A—C11A—C12A	109.2 (6)	C14B—C13B—H13F	109.5
C16A—C11A—C10A	108.1 (5)	C12B—C13B—H13F	109.5
C12A—C11A—C10A	112.5 (5)	H13E—C13B—H13F	108.1
C16A—C11A—H11A	109.0	C15B—C14B—C13B	112.1 (5)
C12A—C11A—H11A	109.0	C15B—C14B—H14E	109.2
C10A—C11A—H11A	109.0	C13B—C14B—H14E	109.2
C11A—C12A—C13A	111.3 (5)	C15B—C14B—H14F	109.2
C11A—C12A—H12A	109.4	C13B—C14B—H14F	109.2
C13A—C12A—H12A	109.4	H14E—C14B—H14F	107.9
C11A—C12A—H12B	109.4	C14B—C15B—C16B	112.7 (5)
C13A—C12A—H12B	109.4	C14B—C15B—H15E	109.1
H12A—C12A—H12B	108.0	C16B—C15B—H15E	109.1
C14A—C13A—C12A	110.6 (7)	C14B—C15B—H15F	109.1
C14A—C13A—H13A	109.5	C16B—C15B—H15F	109.1
C12A—C13A—H13A	109.5	H15E—C15B—H15F	107.8
C14A—C13A—H13B	109.5	C11B—C16B—C15B	110.6 (4)
C12A—C13A—H13B	109.5	C11B—C16B—H16E	109.5
H13A—C13A—H13B	108.1	C15B—C16B—H16E	109.5
C13A—C14A—C15A	110.8 (7)	C11B—C16B—H16F	109.5
C13A—C14A—H14A	109.5	C15B—C16B—H16F	109.5
C15A—C14A—H14A	109.5	H16E—C16B—H16F	108.1
C13A—C14A—H14B	109.5	C9A—N1A—C6A	127.3 (3)
C15A—C14A—H14B	109.5	C9A—N1A—H1	115 (3)
H14A—C14A—H14B	108.1	C6A—N1A—H1	118 (3)
C14A—C15A—C16A	106.3 (10)	C10A—N2A—C9A	128.4 (3)
C14A—C15A—H15A	110.5	C10A—N2A—H2	138 (3)
C16A—C15A—H15A	110.5	C9A—N2A—H2	93 (2)
C14A—C15A—H15B	110.5	C9B—N1B—C6B	125.7 (3)
C16A—C15A—H15B	110.5	C9B—N1B—H3	113 (3)
H15A—C15A—H15B	108.7	C6B—N1B—H3	120 (3)
C11A—C16A—C15A	107.4 (7)	C10B—N2B—C9B	129.1 (3)
C11A—C16A—H16A	110.2	C10B—N2B—H4	119 (3)
C15A—C16A—H16A	110.2	C9B—N2B—H4	112 (3)
C11A—C16A—H16B	110.2	C2A—C1A—H1A	109.5
C15A—C16A—H16B	110.2	C2A—C1A—H1B	109.5
H16A—C16A—H16B	108.5	H1A—C1A—H1B	109.5
C16'—C11'—C10A	146 (3)	C2A—C1A—H1C	109.5
C16'—C11'—C12'	108 (2)	H1A—C1A—H1C	109.5
C10A—C11'—C12'	104.1 (17)	H1B—C1A—H1C	109.5
C16'—C11'—H11'	94.8	O1A—C2A—C1A	107.2 (7)
C10A—C11'—H11'	94.8	O1A—C2A—H2A	110.3
C12'—C11'—H11'	94.8	C1A—C2A—H2A	110.3
C11'—C12'—C13'	106 (2)	O1A—C2A—H2B	110.3
C11'—C12'—H12C	110.6	C1A—C2A—H2B	110.3
C13'—C12'—H12C	110.6	H2A—C2A—H2B	108.5
C11'—C12'—H12D	110.6	C3A—O1A—C2A	118.4 (6)
C13'—C12'—H12D	110.6	C2A'—C1A'—H1'1	109.5
H12C—C12'—H12D	108.8	C2A'—C1A'—H1'2	109.5
C14'—C13'—C12'	116 (4)	H1'1—C1A'—H1'2	109.5
C14'—C13'—H13C	108.3	C2A'—C1A'—H1'3	109.5
C12'—C13'—H13C	108.3	H1'1—C1A'—H1'3	109.5
C14'—C13'—H13D	108.3	H1'2—C1A'—H1'3	109.5
C12'—C13'—H13D	108.3	O1A'—C2A'—C1A'	117 (5)
H13C—C13'—H13D	107.4	O1A'—C2A'—H2'1	108.1
C15'—C14'—C13'	110 (3)	C1A'—C2A'—H2'1	108.0
C15'—C14'—H14C	109.6	O1A'—C2A'—H2'2	108.0
C13'—C14'—H14C	109.6	C1A'—C2A'—H2'2	108.1
C15'—C14'—H14D	109.6	H2'1—C2A'—H2'2	107.3
C13'—C14'—H14D	109.6	C3A—O1A'—C2A'	105 (4)
H14C—C14'—H14D	108.1	C2B—C1B—H1D	109.5
C16'—C15'—C14'	102 (3)	C2B—C1B—H1E	109.5
C16'—C15'—H15C	111.4	H1D—C1B—H1E	109.5
C14'—C15'—H15C	111.4	C2B—C1B—H1F	109.5
C16'—C15'—H15D	111.4	H1D—C1B—H1F	109.5
C14'—C15'—H15D	111.4	H1E—C1B—H1F	109.5
H15C—C15'—H15D	109.2	O1B—C2B—C1B	104.7 (14)
C11'—C16'—C15'	127 (3)	O1B—C2B—H2D	110.8
C11'—C16'—H16C	105.6	C1B—C2B—H2D	110.8
C15'—C16'—H16C	105.6	O1B—C2B—H2E	110.8
C11'—C16'—H16D	105.6	C1B—C2B—H2E	110.8
C15'—C16'—H16D	105.5	H2D—C2B—H2E	108.9
H16C—C16'—H16D	106.1	C3B—O1B—C2B	111.4 (8)
C4B—C3B—C8B	119.7 (4)	C2B'—O1B'—C3B	106.7 (14)
C4B—C3B—O1B	106.5 (6)	C2B'—C1B'—H1G	109.5
C8B—C3B—O1B	133.8 (6)	C2B'—C1B'—H1H	109.5
C4B—C3B—O1B'	145.2 (7)	H1G—C1B'—H1H	109.5
C8B—C3B—O1B'	95.1 (6)	C2B'—C1B'—H1I	109.5
C5B—C4B—C3B	120.5 (5)	H1G—C1B'—H1I	109.5
C5B—C4B—H4B	119.7	H1H—C1B'—H1I	109.5
C3B—C4B—H4B	119.7	O1B'—C2B'—C1B'	109 (3)
C4B—C5B—C6B	120.2 (4)	O1B'—C2B'—H2F	109.8
C4B—C5B—H5B	119.9	C1B'—C2B'—H2F	109.8
C6B—C5B—H5B	119.9	O1B'—C2B'—H2G	109.8
C7B—C6B—C5B	119.6 (4)	C1B'—C2B'—H2G	109.8
C7B—C6B—N1B	118.5 (4)	H2F—C2B'—H2G	108.3
			
C8A—C3A—C4A—C5A	−0.7 (7)	O1B—C3B—C8B—C7B	174.8 (6)
O1A—C3A—C4A—C5A	178.3 (5)	O1B'—C3B—C8B—C7B	178.5 (7)
O1A'—C3A—C4A—C5A	−171 (4)	C6B—C7B—C8B—C3B	0.5 (7)
C3A—C4A—C5A—C6A	−0.4 (7)	O2B—C10B—C11B—C12B	−86.4 (5)
C4A—C5A—C6A—C7A	1.6 (7)	N2B—C10B—C11B—C12B	92.4 (5)
C4A—C5A—C6A—N1A	178.7 (4)	O2B—C10B—C11B—C16B	38.1 (6)
C5A—C6A—C7A—C8A	−1.7 (7)	N2B—C10B—C11B—C16B	−143.2 (4)
N1A—C6A—C7A—C8A	−178.6 (4)	C10B—C11B—C12B—C13B	180.0 (4)
C4A—C3A—C8A—C7A	0.6 (8)	C16B—C11B—C12B—C13B	55.4 (6)
O1A—C3A—C8A—C7A	−178.2 (5)	C11B—C12B—C13B—C14B	−54.8 (7)
O1A'—C3A—C8A—C7A	175 (2)	C12B—C13B—C14B—C15B	54.1 (8)
C6A—C7A—C8A—C3A	0.6 (8)	C13B—C14B—C15B—C16B	−54.3 (7)
O2A—C10A—C11A—C16A	−73.2 (7)	C12B—C11B—C16B—C15B	−53.8 (6)
N2A—C10A—C11A—C16A	108.9 (7)	C10B—C11B—C16B—C15B	−177.5 (4)
O2A—C10A—C11A—C12A	47.5 (8)	C14B—C15B—C16B—C11B	53.7 (7)
N2A—C10A—C11A—C12A	−130.4 (6)	N2A—C9A—N1A—C6A	−178.0 (3)
C16A—C11A—C12A—C13A	−59.9 (9)	S1A—C9A—N1A—C6A	0.3 (6)
C10A—C11A—C12A—C13A	−180.0 (6)	C7A—C6A—N1A—C9A	−47.9 (6)
C11A—C12A—C13A—C14A	56.1 (10)	C5A—C6A—N1A—C9A	135.1 (4)
C12A—C13A—C14A—C15A	−58.2 (13)	O2A—C10A—N2A—C9A	−4.3 (7)
C13A—C14A—C15A—C16A	61.0 (13)	C11'—C10A—N2A—C9A	−168.6 (14)
C12A—C11A—C16A—C15A	63.0 (11)	C11A—C10A—N2A—C9A	173.6 (4)
C10A—C11A—C16A—C15A	−174.2 (8)	N1A—C9A—N2A—C10A	4.1 (6)
C14A—C15A—C16A—C11A	−63.5 (11)	S1A—C9A—N2A—C10A	−174.4 (3)
O2A—C10A—C11'—C16'	−63 (7)	N2B—C9B—N1B—C6B	−175.4 (3)
N2A—C10A—C11'—C16'	101 (7)	S1B—C9B—N1B—C6B	2.4 (6)
O2A—C10A—C11'—C12'	95 (3)	C7B—C6B—N1B—C9B	128.5 (4)
N2A—C10A—C11'—C12'	−101 (3)	C5B—C6B—N1B—C9B	−53.5 (6)
C16'—C11'—C12'—C13'	−14 (5)	O2B—C10B—N2B—C9B	−9.1 (7)
C10A—C11'—C12'—C13'	179 (3)	C11B—C10B—N2B—C9B	172.1 (4)
C11'—C12'—C13'—C14'	63 (5)	N1B—C9B—N2B—C10B	8.7 (6)
C12'—C13'—C14'—C15'	−52 (6)	S1B—C9B—N2B—C10B	−169.2 (3)
C13'—C14'—C15'—C16'	−4 (5)	C4A—C3A—O1A—C2A	−173.4 (6)
C10A—C11'—C16'—C15'	105 (7)	C8A—C3A—O1A—C2A	5.5 (10)
C12'—C11'—C16'—C15'	−52 (6)	C1A—C2A—O1A—C3A	174.4 (6)
C14'—C15'—C16'—C11'	63 (5)	C4A—C3A—O1A'—C2A'	−19 (7)
C8B—C3B—C4B—C5B	2.8 (8)	C8A—C3A—O1A'—C2A'	170 (4)
O1B—C3B—C4B—C5B	−175.7 (5)	C1A'—C2A'—O1A'—C3A	178 (6)
O1B'—C3B—C4B—C5B	179.8 (12)	C4B—C3B—O1B—C2B	174.2 (6)
C3B—C4B—C5B—C6B	0.5 (7)	C8B—C3B—O1B—C2B	−4.0 (10)
C4B—C5B—C6B—C7B	−3.2 (7)	C1B—C2B—O1B—C3B	−173.5 (11)
C4B—C5B—C6B—N1B	178.8 (4)	C4B—C3B—O1B'—C2B'	5 (2)
C5B—C6B—C7B—C8B	2.7 (7)	C8B—C3B—O1B'—C2B'	−177.8 (11)
N1B—C6B—C7B—C8B	−179.2 (4)	C3B—O1B'—C2B'—C1B'	172.4 (19)
C4B—C3B—C8B—C7B	−3.2 (8)		

### Hydrogen-bond geometry (Å, º)

Cg1 is the centroid of the C3*A*–C8*A* ring.

**Table:**

*D*—H···*A*	*D*—H	H···*A*	*D*···*A*	*D*—H···*A*
C2*B*—H2*D*···O2*B*^i^	0.97	2.57	3.264 (10)	128
N1*B*—H3···O2*B*	0.86 (2)	1.91 (3)	2.641 (4)	142 (4)
N1*A*—H1···O2*A*	0.87 (2)	1.90 (3)	2.628 (4)	140 (4)
N2*B*—H4···S1*A*^ii^	0.84 (2)	2.68 (2)	3.469 (3)	157 (3)
N2*A*—H2···S1*B*^iii^	0.85 (2)	2.73 (3)	3.430 (3)	140 (3)
C12′—H12*C*···*Cg*1^iv^	0.90	2.49 (2)	3.42 (1)	159

Symmetry codes: (i) −*x*−1, −*y*+2, −*z*; (ii) *x*, *y*, *z*−1; (iii) *x*, *y*, *z*+1; (iv) −*x*+1, −*y*+1, −*z*+2.
