# Supplementary figures and images for: Crystal structure of N-[(4-eth­oxy­phen­yl)carbamo­thio­yl]cyclo­hexa­ne­carboxamide
Source: Acta Crystallogr E Crystallogr Commun. 2015 Oct 7;71(Pt 11):o820–1. doi: 10.1107/S205698901501806X (PMC4645074; doi:10.1107/S205698901501806X)

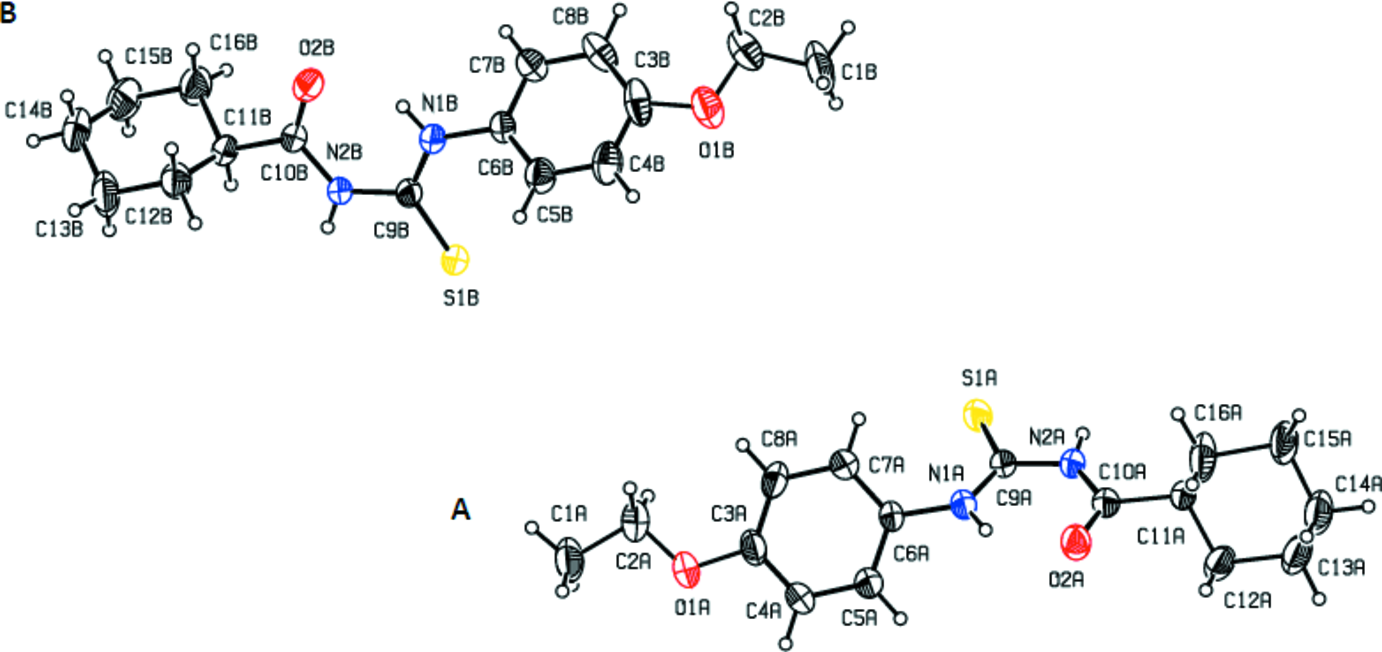

Supplement: Supplementary file 4 [file e-71-0o820-fig1.tif]

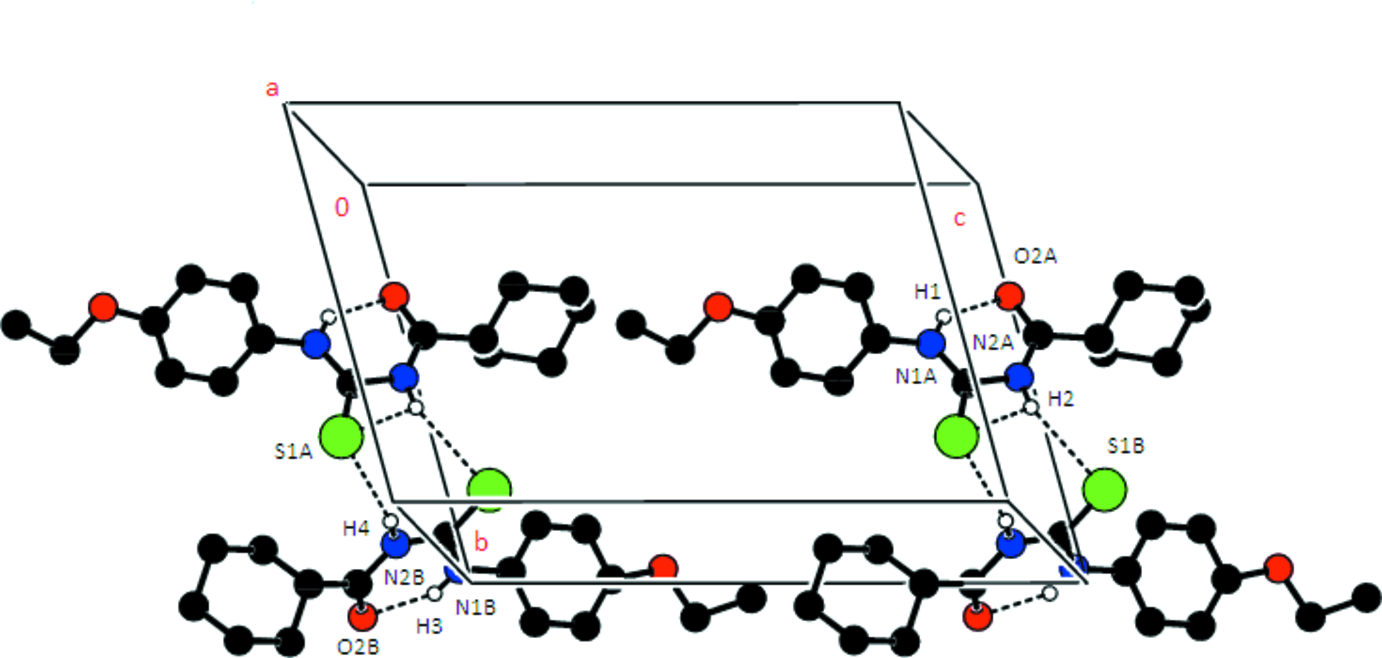

Supplement: Supplementary file 5 [file e-71-0o820-fig2.tif]
